# Group B streptococci vaginal colonization and drug susceptibility pattern among pregnant women attending in selected public antenatal care centers in Addis Ababa, Ethiopia

**DOI:** 10.1186/s12884-018-1791-4

**Published:** 2018-05-04

**Authors:** Solomon Assefa, Kassu Desta, Tsehaynesh Lema

**Affiliations:** 1 Ethiopian Public Health Institute, P.O. Box 1242, Arbegnoch Street, Addis Ababa, Ethiopia; 2 Department of Medical Laboratory Sciences, School of Allied Health Sciences, College of Health Sciences, Addis Ababa University, P.O. Box 1176, Addis Ababa, Ethiopia; 3 Armauer Hansen Research Institute, P.O. Box 1005, Jimma Road, Addis Ababa, Ethiopia; 4 All Africa Leprosy, Tuberculosis, Rehabilitation and Training Center, P.O. Box 165, Jimma Road, Addis Ababa, Ethiopia

**Keywords:** Antimicrobial susceptibility testing, Group B streptococci, Prevalence, Risk factors

## Abstract

**Background:**

Group B Streptococcus (GBS) is the leading cause of septicemia, meningitis, and pneumonia in neonates. Maternal colonization with GBS is the principal risk factor for early-onset disease in infants. Group B Streptococcus is now an important cause of maternal and neonatal morbidity and mortality in many parts of the world. In Ethiopia, few studies have been done on GBS colonization among pregnant women. The aim of this study was to determine the prevalence of GBS colonization, antimicrobial susceptibility patterns and assess risk factors among pregnant women.

**Methods:**

A prospective cross-sectional study was conducted from May to August 2014 at selected public antenatal care (ANC) centers in Addis Ababa, Ethiopia. Clinical and socio-demographical data were collected using structured questionnaire after obtaining written informed consent. A total of 281 lower vaginal swabs were collected and inoculated into 1 ml Todd Hewitt Broth supplemented with gentamicin and nalidixic acid to prevent the growth of contaminants. After overnight incubation, all broths were subcultured on 5% sheep blood agar for isolation of GBS. Antimicrobial susceptibility testing was performed according to the criteria of the Clinical and Laboratory Standard Institute (CLSI) guidelines 2013 by disk diffusion method. Data were entered and analysed using SPSS version 20.0 software. Chi-square test and binary logistic regression analysis were used. *P*-value < 0.05 was considered statistically significant.

**Results:**

The overall prevalence of GBS colonization among pregnant women was 14.6% (41/281). Group B Streptococcus colonization was significantly associated with health institutions (*P* < 0.05). All GBS isolates were susceptible to chloramphenicol. Resistance to tetracycline, cefotaxime, clindamycin, penicillin, vancomycin, ampicillin and erythromycin was 90.2%, 34.1, 26.8%, 19.5, 17%, 14.6 and 7.5% respectively. Multidrug resistance (MDR) (≥ 2 drugs) was detected in 43.9% (18/41) of the isolates.

**Conclusion:**

There was a high frequency of GBS colonization (14.6%) and resistance to the commonly used antibiotics which suggests the importance of the screening of GBS colonization in pregnant women at 35–37 weeks of gestation and testing their antimicrobial susceptibilities in order to provide antibiotic prophylaxis and minimize newborn infection and co-morbidity.

## Background

Group B Streptococcus emerged as the leading cause of neonatal morbidity and mortality in the United States in the 1970s [1–3], with a frequency of 2–3 cases per 1000 live vertical transmission births [4] and case-fatality ratios as high as 50% [3]. Group B Streptococcus is one of the most important causes of neonatal sepsis, meningitis, and pneumonia [5–7]. Group B Streptococcus neonatal infection could be early-onset GBS disease (EOGBSD), which occurs within the first week of life, and late-onset GBS disease (LOGBSD), which occurs between one week to 3 months of age [4, 5].

During pregnancy, approximately 10–30% of women are colonized with GBS in vagina [8, 9] and 60% of their infants acquire this organism through birth canal [6]. Maternal colonization with GBS in the genitourinary or gastrointestinal tract and transmission to the infant during the labor and delivery process is the principal risk factor for early-onset invasive GBS disease [2, 3, 9].

The widespread use of intrapartum antibiotic prophylaxis to prevent early-onset GBS disease has raised concern about the development of antibiotic resistance among GBS isolates [3]. In the absence of a licensed GBS vaccine [3], universal screening of mothers for vaginal or rectal GBS colonization at 35 to 37 weeks of gestation and selective intrapartum antibiotic prophylaxis (IAP) for all screen-positive women is the strategy currently recommended to reduce incidence of colonization in neonates and prevent early-onset GBS-related diseases [3, 9, 10].

Group B Streptococcus is now recognized to be an important cause of maternal and neonatal morbidity and mortality in many parts of the world [6, 7]; however, it has been little studied in Ethiopia [11, 12]. Therefore, this study was conducted to determine the prevalence of GBS colonization, antimicrobial susceptibility pattern and assess risk factors related to GBS among pregnant women attending in selected public ANC centers at Addis Ababa, Ethiopia.

## Methods

### Study design and setting

A prospective cross-sectional study was conducted among pregnant women attending ANC clinics of ALERT Center, Alem Bank, and Woreda 03 health centers, Addis Ababa, Ethiopia from May to August 2014. ALERT Center is one of the specialized tertiary referral hospitals in the country. While the two health centers are mainly engaged in routine antenatal care and delivery service in addition to other health routine care deliveries.

### Study population, sample size, and sampling technique

A total of 281 pregnant women (from 35 to 37 weeks of gestation) attending the routine ANC follow up were screened for GBS colonization at ALERT Center (*n* = 141), Alem Bank (*n* = 88) and Woreda 03 (*n* = 52) Health centers. Eligible study participants were enrolled in this study using consecutive sampling technique. The sample size was calculated based on the prevalence indicated in the previous study using single population proportion formula [12]. Expected margin of error (d) was 0.05 and confidence interval (z) was 95%. Contingency for the unknown circumstance was 10%. Pregnant women with a premature rupture of membranes (PROM) and history of antibiotic(s) use within two weeks prior to recruitment were excluded from this study.

### Data collection

After obtaining written informed consent, socio-demographic and clinical data were collected using a structured questionnaire. Moreover, recent HIV result was taken from study participants’ medical records. Data were collected by well-trained gynaecologist and midwives.

### Specimen collection and transport

Vaginal swabs were taken from the lower vagina using sterile cotton swab according to the Center for Disease Control and Prevention (CDC) and American College of Obstetricians and Gynecologists (ACOG) guidelines [3, 13], and inoculated directly into Todd-Hewitt broth (THB) (Oxoid Ltd., Basingstoke, Hampshire, England) and immediately transported to the Microbiology Laboratory of ALERT Center for further analysis.

### Culture and identification of group B streptococci

The vaginal swabs were placed into 1 ml THB (Oxoid Ltd., Basingstoke, Hampshire, England) supplemented with gentamicin (8 μg/ml) (Intas pharmaceutical Ltd., Matoda village, Ahmedabad, Gujarat, India) and nalidixic acid (15 μg/ml) (Sigma Aldrich, Italy) to prevent growth of contaminants [3]. The broth was incubated for 18–24 h at 35–37 °C and inoculated on 5% sheep blood agar (SBA) (Oxoid Ltd., Basingstoke, Hampshire, England) and incubated overnight in 5% CO_2_ atmosphere for 18–24 h. Broth cultures showing no visible turbidity after overnight incubation were re-incubated for additional hours and then subcultured after 48 h on SBA. Suspected GBS colonies (pink colonies, with narrow beta-hemolysis) were confirmed by Gram stain, catalase test and Christie, Atkins, and Munch-Peterson (CAMP) test.

### Antimicrobial susceptibility testing

Antimicrobial Susceptibility Testing was performed according to Clinical and Laboratory Standard Institute guidelines (CLSI) guidelines 2013 using Kirby-Bauer disk diffusion method [14]. Direct colony suspension in sterile saline, equivalent to 0.5 McFarland standard was done and inoculated on Muller-Hinton agar (MHA) (Oxoid Ltd., Basingstoke, Hampshire, England) with 5% sheep’s blood using a sterile cotton swab. An antibiotic disk (Oxoid Ltd., Basingstoke, Hampshire, England) was placed on the agar with clindamycin and erythromycin disks placed 16 mm from each other in order to detect inducible resistance to clindamycin (D-zone test) and incubated at 35–37 °C with 5% CO_2_ atmosphere for 18–24 h. The zone of growth inhibition was measured using the Oxoid ruler (Oxoid Ltd., Basingstoke, Hampshire, England) and Linex ruler (Linex 116, Denmark). The antibiotics used were penicillin (P) (10 μg), ampicillin (AMP) (10 μg), erythromycin (E) (15 μg), clindamycin (DA) (2 μg), vancomycin (30 μg), cefotaxime (CTX) (30 μg), chloramphenicol(C) (30 μg) and tetracycline (TE) (30 μg). The result was interpreted according to CLSI guidelines 2013 as susceptible, intermediate or resistant [14].

### Quality control

The sterility of culture media was checked by incubating overnight at 35–37 °C without specimen inoculation. *Enterococcus faecalis* (ATCC 29212), *S. agalactiae* (ATCC 27956), *S. pyogenes* (ATCC 19615), *S. aureus* (ATCC 25923) and *E. coli* (ATCC 25922) strains were used as a quality control organisms for culture and antimicrobial susceptibility testing.

### Data analysis

Data were coded, entered, cleaned and analyzed by using SPSS version 20.0 software. Frequency distribution, percentage, tables, and charts were used to present results. Explanatory variables were individually cross-tabulated with the outcome variable and statistical significance assessed using chi-square and logistic regression model. A *p*-value less than 0.05 was considered statistically significant.

### Ethical consideration

This study was approved and ethically cleared by the Departmental Ethics and Research Committee (DERC) of Department of Medical Laboratory Science, College of Health Science, Addis Ababa University (AAU) (Ref.No.: MLS/501/14and protocol No.: DRERC 056/13/MLS), Armauer Hansen Research Institute / All African Leprosy, Tuberculosis, Rehabilitation and Training Center (AHRI/ALERT) Ethical Review Committee (AAERC) (Project Reg. No.:PO17/14), Institutional Review Board (IRB) of Addis Ababa Health Bureau (Ref.No.: AAHB/5900/227) and National Research Ethics Review Committee (NRERC) (Ref.No.: 3.10/796/06). Official permission from the study sites was obtained. Written informed consent was obtained from each study participant. All participants’ results were kept confidentially.

## Results

### Socio-demographic characteristics

A total of two hundred eighty-one (281) pregnant women (from 35 to 37 weeks of gestation) were enrolled from May to August 2014. The age of the study participants ranged from 18 to 39 years with a mean age of 26.46 (± 4.41) years. Most of the study the participants were between the ages of 25–29 years 120 (42.7%). Most of the study participants were married (97.5%). The majority of the study participants were housewives (68.3%) (Table 1).

**Table 1:** Socio-demographic characteristics of pregnant women investigated for GBS at three health institutions (*n* = 281)

Socio-demographic characteristics	Frequency	Percentage
Health Institutions		
ALERT Center	141	50.2%
Woreda 03 Health Center	52	18.5%
Alem Bank Health Center	88	31.3%
Age groups		
15–19	8	2.8%
20–24	85	30.2%
25–29	120	42.7%
30–34	49	17.4%
≥ 35	19	6.8%
Marital status		
Married	274	97.5%
Single	4	1.4%
Divorced	2	0.7%
Widowed	1	0.4%
Occupation		
Civil Servant	35	12.5%
House Wives	192	68.3%
Business Women	54	19.2%

### Group B Streptococcus colonization

The overall prevalence of GBS colonization among pregnant women at 35–37 weeks of gestation was 14.6% (41/281). The prevalence of GBS in the three health institutions was 20 (22.7%), 17 (12.1%) and 4 (7.7%) in Alem Bank health center, ALERT center, and Woreda 03 health center respectively.

### Antimicrobial susceptibility testing

The antimicrobial susceptibility testing results of GBS isolates are summarized in Table 2. All GBS isolates were 100% susceptible to chloramphenicol. Most isolates (80.5% to 92.5%) were susceptible to penicillin G, vancomycin, ampicillin, and erythromycin. Most GBS isolates (90.2%) were resistant to tetracycline (Table 2).

**Table 2:** Antimicrobial susceptibility pattern of GBS isolates from pregnant women recruited from three health institutions (*n* = 41)

Antibiotics	Disk Potency (μg)	Susceptible	Intermediate	Resistant
Chloramphenicol (C)	30	41(100%)	0	0
Erythromycin (E)	15	38(92.5%)	0	3(7.5%)
Ampicillin (AMP)	10	35(85.4%)	0	6(14.6%)
Vancomycin (VA)	30	34(83%)	0	7(17%)
Penicillin G (P)	10	33(80.5%)	0	8(19.5%)
Clindamycin (DA)	2	30(73.2%)	0	11(26.8%)
Cefotaxime (CTX)	30	27(65.9%)	0	14(34.1%)
Tetracycline (TE)	30	3(7.3%)	1(2.4%)	37(90.2%)

### Multi-drug resistance pattern

Multidrug resistance (MDR) (≥ 2 drugs) was detected in 43.9% (18/41) of the isolates. Resistance to 2, 3, 4, 5 and 6 drugs was found to be 38.88%, 5.55, 27.77%, 11.11 and 16.66% respectively (Table 3).

**Table 3:** Multi-drug resistance pattern of GBS isolated from pregnant women recruited from three health institutions (*n* = 41)

Drugs resistance pattern (Antibiogram)	No. of drug to which strains were resistant	No. of resistant strains (%)
CTX:TE	2	2 (11.11)
DA:TE	2	1 (5.55)
E: TE	2	1 (5.55)
TE: VA	2	3 (16.66)
CTX: DA: TE	3	1 (5.55)
AMP: CTX: E: TE	4	1 (5.55)
CTX: DA: P: TE	4	2 (11.11)
CTX:DA:P:VA	4	1 (5.55)
CTX:DA:TE:VA	4	1 (5.55)
AMP: CTX: DA: P:TE	5	2 (11.11)
AMP: CTX: DA: E: P: TE	6	1 (5.55)
AMP:CTX: DA: P: TE: VA:	6	2 (11.11)

*TE* Tetracycline, *CTX* Cefotaxime, *VA* Vancomycin, *DA*: Clindamycin, *E* Erythromycin, *P* Penicillin G, *AMP* Ampicillin

### Risk factors for group B streptococci

#### Socio-demographic factors

The association of socio-demographic variables with GBS colonization is summarized in Table 4. In univariate analysis, GBS colonization showed statistically significant association with health institutions (*P* < 0.05). However, there was no statistically significant association between GBS colonization and age group, marital status and occupation (*P* > 0.05). This study revealed a higher GBS colonization rate among pregnant women of age group 15–19 years (25%) than the age group ≥35 years (10.5%). However, the difference was not statistically significant (P > 0.05). In multivariable logistic regression analysis, GBS colonization was significantly associated with health institutions (P < 0.05) (Table 4).

**Table 4:** Association between socio-demographic factors and GBS colonization among pregnant women at three health institutions (*n* = 281)

Socio demographic factors	Total	GBS Culture		COR (95% C.I)	*p*-value ^a^	AOR(95% C.I)	*p*-value^b^
		GBS negative N (%)	GBS positive N (%)				
Age group							
15–19	8	6(75)	2(25)	2.83(0.32–24.8)	0.35		
20–24	85	72(84.7)	13(15.3)	1.54(0.32–7.45)	0.59		
25–29	120	102(85)	18(15)	1.50(0.32–7.06)	0.61		
30–34	49	43(87.8)	6(12.2)	1.19(0.22–6.47)	0.84		
≥ 35	19	17(89.5)	2(10.5)	1			
Health Institutions							
ALERT Center	141	124(87.9)	17(12.1)	1		1	
Woreda 03 HC	52	48(92.3)	4(7.7)	0.61(0.20–1.90)	0.392	0.71 (0.22–2.27)	0.568
Alem Bank HC	88	68(77.3)	20(22.7)	2.15(1.05–4.37)	0.035*	2.63 (1.22–5.65)	0.013*
Marital Status							
Married	274	234(85.4)	40(14.6)	1			
Single	4	3(75)	1(25)	1.95(0.20–19.2)	0.57		
Divorced	2	2(100)	0(0.0)	0.000	0.99		
Widowed	1	1(100)	0(0.0)	0.000	1.00		
Occupation							
Civil servant	35	32(91.4)	3(8.6)	1			
House wife	192	163(84.9)	29(15.1)	1.9(0.55–6.61)	0.314		
Business women	54	45(83.3)	9(16.7)	2.13(0.54–8.51)	0.283		

*Significant at *p*-value< 0.05; ^1^ indicates logical reference group or constant; *CI* Confidence Interval, *COR* Crude odds ratio, *AOR* Adjusted odds ratio, ^a^ *p*-value obtained by binary; ^b^ *p*-value obtained by multiple logistic regression; HC: Health Center; ^N^ Number; ^%^ Percentage

#### Obstetric factors

In univariate analysis, GBS colonization did not show statistically significant association with the number of antenatal visits, gravidity, history of spontaneous abortion and stillbirth (*P* > 0.5). Pregnant women with no history of stillbirth (14.9%) showed higher GBS colonization rates than those mothers with a history of stillbirth (7.7%). However, the difference was not statistically significant (*P* > 0.05) (Table 5).

**Table 5:** Association between obstetric factors and GBS colonization among pregnant women at three health institutions (*n* = 281)

Obstetric Factors	Total	GBS culture		COR (95% C.I)	*p*-value^a^	AOR (95% C.I)	*p*-value^b^
		GBS negative N (%)	GBS positive N (%)				
ANC visit							
One times	6	5(83.3)	1(16.7)	1.12 (0.13–10.1)	0.917		
Two times	37	32(86.5)	5(13.5)	0.88 (0.31–2.51)	0.808		
Three times	99	85(85.9)	14(14.1)	0.93 (0.45–1.92)	0.836		
Four times	139	118(85.4)	21(14.6)	1			
Type of Gravida							
Primigravida	88	74(84.1)	14(15.9)	1.16 (0.58–2.35)	0.673		
Multigravida	193	166(86)	27(14)	1			
Still birth							
No	268	228(85.1)	40(14.9)	1			
Yes	13	12(92.3)	1(7.7)	0.48 (0.06–3.76)	0.480		
Abortion							
No	211	180(85.3)	31(14.7)	1			
Yes	70	60(85.7)	10(14.3)	0.97 (0.45–2.09)	0.934		
HIV Infection							
No	256	221(86.3)	35 (13.7)	1		1	
Yes	25	19 (76)	6 (24)	1.99 (0.75–5.34)	0.170	2.8 (0.97–8.11)	0.057

^1^logical reference; *CI* Confidence interval, *COR* Crude odds ratio, *AOR* Adjusted odds ratio, ^N^ Number; ^%^ Percentage; ^a^ *p*-value obtained by binary; ^b^ *p*-value obtained by multiple logistic regression; Adjusted for HIV status

### HIV infection

Of 281 pregnant women screened for GBS colonization, 25 (8.9%) were HIV positive. Among the pregnant women with HIV infection, 6 (24%) were positive for GBS and among pregnant women with HIV negative, 35 (13.7%) were GBS positive. However, this difference was not statistically significant (P > 0.05) (Table 5). The Confounding effect of HIV infection was checked as its biological plausibility is expected with GBS colonization, but statistically failed to be found independently significantly associated.

## Discussion

In this study, the overall prevalence of GBS among pregnant women was 14.6%. This finding was almost similar with reports from other developing countries; in Malawi (16.5%) and Nigeria (11.3%) [15, 16], but higher than those reported in Gondar, North Ethiopia (9%) and Mozambique (1.8%) [11, 17] and slightly lower than those reported in Hawassa, South Ethiopia (20.8%), Tanzania (23%) and Zimbabwe (21%) [6, 12, 18]. The finding of this study was also comparable to the studies done in some European countries; in North-Eastern Italy (17.9%) by Busetti M et al., Turin, Italy (18%) by Savoia D et al., and Poland (17.2%) [19–21], but higher than those reported in Northern Greece (6.6%) [22], and slightly lower than those reported in Switzerland (21%), UK (21.3%) and Netherlands (21%) [23–25].

The result of our study was also almost similar with the studies conducted in Texas, USA (12.2%) [26], and in Brazil ranging from 14.6% to 20.4 [27–29], but higher than those reported in Argentina (7.6%) [30].The finding of this study (14.6%) was higher than reports from some Asian countries; such as Taiwan (6.2%), India (2.3%), China (7.1%) and Korea (8.3%) [31–34]. The variations between countries could possibly be due to differences in the sample size and type of sites cultured, culture methods, socio-economic status, sexual behavior and geographic areas.

In this study, GBS colonization was significantly associated with health institutions (*P* < 0.05). This finding was consistent with reports from another study [34]. Those pregnant women who were managed in Alem Bank Health center were 2.6 times more likely to be colonized with GBS compared to those pregnant women who were managed in the ALERT center (AOR: 2.6, 95% C.I: 1.22–5.65). This difference might be due to specimen collection techniques of health care providers. Those providers in the centers with lower GBS rates may not have used proper hygienic sampling techniques including insertion of the swab into the vaginal. Therefore, it needs further investigation to confirm the relationship between GBS colonization and health institutions.

Group B Streptococcus colonization in this study was higher in HIV infected pregnant women, though the association was not significant. GBS colonization and HIV infection (*P* > 0.05). This might probably be due to the small number of HIV infected pregnant women among the studied population. Similar findings have been reported in studies conducted in Tanzania and Malawi [6, 35]. However, a study conducted in the Democratic Republic of Congo, colonization rate was significantly associated with HIV infection [36].

In the present study, we observed that primigravida women were more often associated with GBS colonization, though it was not statistically significant (*P* > 0.05). Similar findings have been reported in Ethiopia (2012), Nigeria and Brazil [12, 15, 28]. However, in another study colonization rates were found to be significantly greater among multigravida women than primigravida women (*P* < 0.001) [32, 34, 37]. This might be due to geographical variation. Therefore, further studies are needed to confirm the correlation between gravidity and colonization by GBS from the different geographical location.

In the present study, history of spontaneous abortion did not influence GBS colonization in pregnant women. Similar findings have been reported in studies conducted in Tanzania and India [6, 32]. However, in another studies history of spontaneous abortion showed significant association with GBS colonization [34, 36]. Therefore, further studies are needed to confirm the correlation between abortion and colonization by GBS. The previous history of stillbirth did not influence GBS colonization. The lack of association with this factor might be explained by the fact that the numbers of participants in this study with such risk factor were small. This finding was consistent with studies from Dar es Salaam, Tanzania (2009) by Joachim A et al. [6].

Penicillin and ampicillin are the drugs of choice for prevention or treatment of GBS infections, and clindamycin and erythromycin are the recommended alternatives for patients who are allergic to ß-lactam agents. The widespread use of these antibiotics to prevent early-onset GBS disease has raised concern about the development of antibiotic resistance among GBS isolates [3].

In this study, all GBS isolates were susceptible to chloramphenicol. In our study, we observed resistance to penicillin (19.5%) and ampicillin (14.6%) which are the first choice of drugs for intrapartum prophylaxis. This did not match with the CDC 2010 guidelines study, which did not find any resistance to penicillin. These findings were comparable to those reported in another study [15]. The expanded use of beta-lactam antimicrobials in the treatment of several infective clinical syndromes and the free accessibility of purchase over the counter might be the cause of the emergence of GBS resistance strains in this environment.

The CDC 2010 guideline recommends testing of GBS isolates for susceptibility to clindamycin and erythromycin, as they are the drugs of choice for penicillin-allergic women at high risk for anaphylaxis [3]. An increase in resistance of GBS to erythromycin has been reported [1, 15, 38–41]. In this study, we found that 7.5% of the isolates were resistant to erythromycin. This was consistent with reports from other studies [6, 7, 12, 42, 43]. This rate of erythromycin resistance in the GBS isolates strongly supports the current CDC recommendation that antibiotic susceptibility test should be performed if erythromycin therapy is needed to prevent neonatal GBS infection. With respect to resistance to clindamycin, the finding of this study (26.8%) was similar to those reports from other studies [6, 15, 38–41, 43, 44]. Since clindamycin is another alternative antibiotic recommended by the CDC for pregnant women who are allergic to penicillin, the resistance level underline the need of carrying out a susceptibility test. This might be due to the widespread use of the antibiotics.

Vancomycin is recommended for GBS-colonized mothers with a high risk of anaphylaxis to penicillin and if the isolate is resistant to clindamycin [3]. In this study, we found that 17% of the isolates were resistant to vancomycin. This finding was comparable to those reported in another study [15]. Since vancomycin is another alternative drug recommended by the CDC for pregnant women who are allergic to penicillin and clindamycin-resistant isolates, the resistance level underline the need of carrying out a susceptibility test. Most GBS isolates (90.2%) were resistant to tetracycline which showed consistency with reports from other studies [12, 32, 38, 42, 45]. This may probably be due to the widespread use of this antibiotics and ease of procurement of antibiotic and/or could be attributed to the indiscriminate use of antimicrobial drugs in this area. The cefotaxime resistance (34.1%) in this study was difficult to explain since cefotaxime was rarely used in Ethiopia. In contrast to this, high susceptibility to cefotaxime was observed in other studies [15, 42].

## Conclusion

There was high isolation rate of GBS (14.6%) among pregnant women. The resistance of the isolates to the commonly used antibiotics including penicillin, ampicillin, and clindamycin in this study calls for screening of all pregnant women at 35–37 weeks of gestation. Performing susceptibility testing before administration of any of these antibiotics to provide antibiotic prophylaxis to GBS carrier is necessary. The finding of this study also signifies that GBS infection might be a silent clinical problem that is undiagnosed in the present study area, therefore, it requires advocacy work for awareness and concerted effort for preventive measures.

## Abbreviations

ACOG: American college of obstetricians and gynecologists
AHRI: Armauer hansen research institute
ALERT: All african leprosy, tuberculosis, rehabilitation and training center
ANC: Antenatal care
ATCC: American type culture collection
CAMP Test: Christie, atkins, munch, peterson test
CDC: Center for disease control and prevention
CLSI: Clinical and laboratory standard institute
DRERC: Departmental research and ethics review committee
EOGBSD: Early-onset group B streptococcal disease
GBS: Group B streptococci
HIV: Human immunodeficiency virus
IAP: Intrapartum antibiotics prophylaxis
IRB: Institutional review board
LOGBSD: Late- onset group B streptococcal disease
MHA: Muller Hinton Agar
NRERC: National research ethics review committee
PROM: Premature rupture of membranes
SBA: Sheep blood agar
SPSS: Statistical package for social sciences
THB: Todd hewitt broth
